# Capacitive Sensors Based on Recycled Carbon Fibre (rCF) Composites

**DOI:** 10.3390/s24144731

**Published:** 2024-07-21

**Authors:** Oliver Ozioko, Daniel C. Odiyi, Uchenna Diala, Fiyinfoluwa Akinbami, Marshal Emu, Mahmoud Shafik

**Affiliations:** 1 Department of Electrical and Electronic Engineering, College of Science and Engineering, University of Derby, Derby DE22 1GB, UK; 2 Department of Mechanical and Manufacturing Engineering, College of Science and Engineering, University of Derby, Derby DE22 1GB, UK; d.odiyi@derby.ac.uk (D.C.O.);

**Keywords:** capacitive soil moisture sensor, recycled carbon fibre composites, capacitive touch sensors

## Abstract

Recycled carbon fibre (rCF) composites are increasingly being explored for applications such as strain sensing, manufacturing of automobile parts, assistive technologies, and structural health monitoring due to their properties and economic and environmental benefits. The high conductivity of carbon and its wide application for sensing makes rCF very attractive for integrating sensing into passive structures. In this paper, capacitive sensors have been fabricated using rCF composites of varying compositions. First, we investigated the suitability of recycled carbon fibre polymer composites for different sensing applications. As a proof of concept, we fabricated five touch/proximity sensors and three soil moisture sensors, using recycled carbon fibre composites and their performances compared. The soil moisture sensors were realised using rCF as electrodes. This makes them corrosion-resistant and more environmental-friendly, compared to conventional soil moisture sensors realised using metallic electrodes. The results of the touch/proximity sensing show an average change in capacitance (ΔC/C~34) for 20 mm and (ΔC/C~5) for 100 mm, distances of a hand from the active sensing region. The results of the soil moisture sensors show a stable and repeatable response, with a high sensitivity of ~116 pF/mL of water in the linear region. These results demonstrate their respective potential for touch/proximity sensing, as well as smart and sustainable agriculture.

## 1. Introduction

Over the last few years, there has been a burgeoning demand for the use of recycled carbon fibres (rCF) [1,2,3] in applications such as automobiles [4], rail [5], aerospace [6], wind turbines [7], and structural health monitoring [8,9]. This was originally motivated by the quest to reduce material cost, but currently, the additional economic benefits that these materials bring, as a result of their unique mechanical strength and recyclability [3,10], are being more widely recognised. Furthermore, their radiolucent property [10] makes them attractive for healthcare applications, where they are being explored for the fabrication of fixation systems [11], which are necessary for the treatment of unstable long bone fractures. The increasing awareness of the environmental benefits of recycled materials also makes them attractive for the fabrication of sustainable sensors. Researchers have previously explored the use of different recyclable materials [12], such as hydrogel [13], Polylactic acid (PLA)-based materials, silk proteins, self-healing, and cellulose-based materials [14,15], for the realisation of sensors. These include pressure [16] and electrochemical sensors for healthcare applications [17,18]. Recently, researchers have also reported the realisation of 3D-printable composite materials, achieved by integrating graphite into the acrylonitrile butadiene styrene (ABS) polymer matrix.

Generally, carbon-based materials (including fibre and nanoparticles) have been widely explored for sensing applications as well as energy harvesting, with applications in numerous areas such as healthcare, robotics, self-sensing concrete, and wearable applications [19,20,21]. In particular, carbon fibres are very strong with very good chemical resistance properties. From a sustainability standpoint, the use of recycled carbon fibre promotes economic circularity. With less dependence on mining to extract new materials, there is less capital expenditure and lower environmental degradation due to pollution [22]. Although researchers have explored the use of other recycled materials for the realisation of sensors [17,23], their adoption in corrosion-prone areas (e.g., monitoring of soil moisture) and intrinsic sensing applications are still limited. More so, the active sensing layer is always different from the passive materials, where they are integrated. This poses integration challenges in some of these applications.

Therefore, the use of rCF is promising for the integration of sensing into passive structures and systems (such as automobile parts, assistive technologies, etc.), which are currently being manufactured using rCF [24]. This means that rather than just having passive structures made with rCF, the sensing properties of rCF could be harnessed to develop different kinds of sensors, such as touch, pressure, proximity, and soil moisture sensors. Currently, most of the reported works on rCF focus on its use for structural applications, especially self-sensing concretes, which primarily harness the piezoresistive property of the integrated rCF for structural health monitoring [8,9]. However, rCF composites also have the potential to be used for other sensing applications due to their multifunctional capabilities [20,25]. For instance, in [26], a multifunctional carbon fibre composite consisting of two carbon fibre (CF)layers embedded in a structural battery electrolyte (SBE) was demonstrated. This structure was shown to sense strain as well as harvest vibration energy. Piezoelectric energy harvesters have also been realised by harnessing the unique properties of CF [27].

rCF can also be utilised as an electrode for the development of soil moisture sensors. Soil moisture sensors [28] commonly measure the moisture content of the soil when its electrodes are inserted into the soil. Such sensors have been explored using electrodes made of different materials (e.g., copper, stainless steel, and gold) and transduction mechanisms (e.g., resistive and capacitive) (See Table 1). In [29], a polymethyl methacrylate (PMMA) coated copper electrode was used to realise a capacitive soil moisture sensor and the performance of various shapes of electrodes was studied. In [30], zirconia was coated on a stainless steel electrode to realise a soil moisture sensor. Zicornia was utilised because of its chemical inertness, which has benefits for sensing soil moisture. One of the key issues faced by such sensors is the corrosion of the metallic electrodes due to their exposure to moisture in the soil. This leads to inaccurate measurements and, eventually, failure of the sensors. However, replacing such metallic electrodes with electrodes made using rCF has the potential to remedy the issue of rust and improve reliability.

A capacitive sensing mechanism is one of the popular transduction mechanisms used due to its simple structure and resistance to temperature drift [35]. More so, capacitive sensors are known to have simple readout electronics [36], making it easy for them to be implemented in several applications. They have been widely explored as the transduction mechanism for a number of sensors, including touch sensors, proximity sensors, soil moisture sensors, tilt sensors, and pressure sensors. These capacitive sensors employ different structures, including coplanar and parallel structures [36], depending on the application. Typically, the structure of a capacitive sensor includes a dielectric material sandwiched between the two parallel conducting plates, and the properties of the sandwiched dielectric material significantly influence the output of the sensors [35]. One such reported applications are the capacitive-based soil moisture sensors [37], where the electrodes of the sensors are made using two conducting materials, and the soil, with its water content, acts as the dielectric layer. Furthermore, a capacitive transduction mechanism has been employed for the realisation of proximity sensors. These types of sensors detect the proximity of objects through a corresponding change in capacitance of the sensor, when an object approaches. Different materials, including metal (e.g., copper, gold, etc.), have been utilised in the fabrication of capacitive-based proximity sensors. Since rCF is conductive, it can be a good alternative in applications where intrinsic sensing is desired. The integration of rCF composites into capacitive sensor technology leverages the inherent electrical conductivity and structural integrity of carbon fibres. These composites exhibit enhanced dielectric properties, enabling the precise detection of changes in capacitance caused by external stimuli, such as pressure, strain, or chemical interactions. Moreover, the durability of rCF composites makes them suitable for diverse sensor configurations and applications.

In this paper, we explored the potential of using recycled carbon fibre as electrodes for the realisation of different kinds of capacitive-based sensors (e.g., touch/proximity sensors and soil moisture sensors). First, rCF laminates, with varying compositions, were fabricated using the Vacuum-Assisted Resin Infusion (VARI) technique to understand the optimal composition for the proposed sensing applications. As a proof of concept, the different rCF laminates were utilised to fabricate five similar capacitive-based touch sensors and three different soil moisture sensors. The different laminates and sensors were characterised, and their results were compared. This rCF-based capacitive soil moisture sensor, presented here, was realised using rCF as electrodes, providing a corrosion-resistant advantage over the metal electrodes typically employed in conventional soil moisture sensors. The results show a relatively high sensitivity and repeatability.

## 2. Materials and Methods

This section presents the steps for the fabrication carried out in this work. These include (1) fabrication of the recycled carbon fibre composites, using different fibre-to-epoxy ratios; (2) fabrication of the capacitive touch sensors (TS); (3) fabrication of the capacitive soil moisture sensors (SMSs).

### 2.1. Fabrication of the Recycled Carbon Fibre Composites

The electrodes used in this work were fabricated using composite materials consisting of recycled carbon fibre reinforcement and epoxy resin. The reinforcement was a 300 gsm non-woven recycled carbon fibre mat. The matrix was composed of IN2 epoxy resin and AT30 slow hardener (Easy Composites Ltd., Stoke-on-Trent, UK), mixed at a ratio of 100:30 by weight. To investigate the relationship between mechanical properties and sensing performance, various fibre-to-resin ratios were employed. This approach allowed for the exploration of a range of compositions and their subsequent effects on sensor sensitivity.

**Figure 1:**
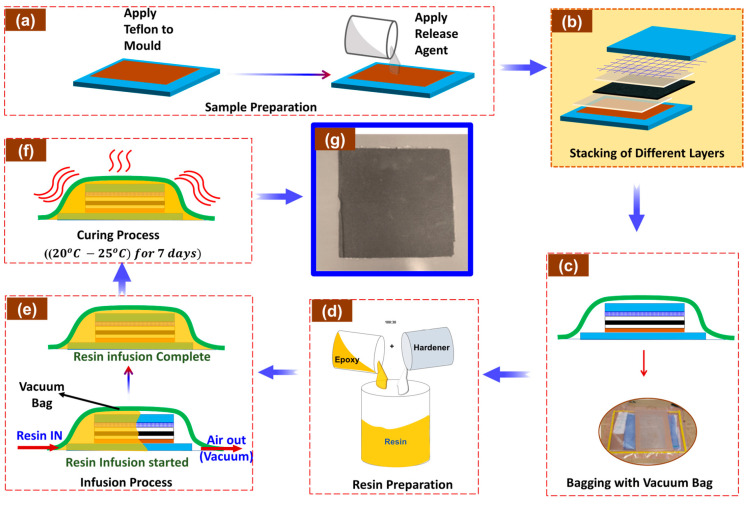
The fabrication scheme for the recycled carbon fibre composites. (**a**) Applying Teflon to the mould. (**b**) stacking of different layers. (**c**) Bagging with vacuum bag. (**d**) Preparation and mixing of resin. (**e**) Infusion process. (**f**) Curing of the sample. (**g**) Image of the fabricated rCF composite.

The manufacturing technique involved vacuum resin infusion, as illustrated in Figure 1. First, non-porous, Polytetrafluoroethylene (PTFE)-coated, woven glass fabric (Teflon) was adhered to an aluminium mould surface, and the surface was cleaned with acetone, followed by multiple applications of coats of release agent to the surface to facilitate removal of the cured laminate, as illustrated in Figure 1a. A configuration of four layers of non-woven recycled carbon fibre mat (200 mm × 200 mm), peel ply, and mesh were stacked on the mould in the order shown in Figure 1b. The set-up was sealed in a vacuum bag, as shown in Figure 1c. Resin feed and vacuum lines were connected to respective ports within the bagged set-up. Drop leak tests were performed to check for leaks and ensure vacuum pressure integrity. Subsequently, the epoxy resin and hardener were mixed (Figure 1d) and degassed. This was followed by the infusion process, which involved connecting a vacuum pump to create negative pressure, causing the resin to flow from the feed line into the dry fibre reinforcement until fully impregnated, as shown in Figure 1e. Upon completion, the set-up was allowed to cure at room temperature for 7 days, as recommended by the supplier (Figure 1f), before demoulding to achieve the cured laminate, as seen in Figure 1g. Three recycled carbon fibre composites, with varying fibre volume fractions, were fabricated by repeating the process outlined in Figure 1.

### 2.2. Determination of Fibre Volume Fraction

The fibre volume fraction (FVF) of the manufactured laminates was determined using matrix digestion, with procedure F, outlined in ASTM D3171-99 [38]. The density of the samples was determined according to ISO 14127:2024 standard [39]. From each of the three laminates, five specimens measuring 10 mm × 10 mm × 10 mm were cut and weighed to 0.0001 g accuracy, then immersed in 50 mL of concentrated nitric acid in a closed high-pressure vessel. The specimens were then digested in an Ethos Up Microwave digestion oven at 120 °C for 80 min. After digestion, the residue recycled carbon fibre was washed and oven-dried at 100 °C for 60 min. The weight of fibre was measured, and the fibre volume fraction (FVF) and porosity volume fraction were computed based on known densities of the composites, fibre, and matrix using the following equations.

Fibre volume content as a percentage ${(V}_{f})$ is determined using Equation (1):

(1) $$V_{f}=\left(\frac{M_{f}}{M_{i}}\right)*100*\left(\frac{\rho_{c}}{\rho_{f}}\right)$$

where

$M_{f}$ = Final mass of the specimen;

$M_{i}$ = Initial mass of the specimen;

$\rho_{c}$ = Density of composite;

$\rho_{f}$ = Density of fibre.

The resin volume content ${(V}_{m})$, as a percentage, is determined using Equation (2):

(2) $$V_{m}=\left(\frac{M_{i}-M_{f}}{M_{i}}\right)*\left(\frac{\rho_{c}}{\rho_{r}}\right)$$

where

$M_{f}$ = Final mass of the specimen;

$M_{i}$ = Initial mass of the specimen;

$\rho_{c}$ = Density of composite;

$\rho_{r}$ = Density of resin.

The void volume content, as a percentage, can be evaluated using Equation (3):

(3) $$V_{v}=100-(V_{f}+V_{m})$$

where

$V_{f}$ = Calculated fibre volume content;

$V_{m}$ = Calculated resin volume content.

### 2.3. Flexural Strength Test

The flexural properties of the specimens were characterised according to BS ISO 178:2019 using a three-point bending test configuration. Rectangular specimens, with dimensions of 80 mm × 10 mm × 4 mm, were tested under a span length of 64 mm using a Tinius Olsen universal testing machine supplied by Tinius Olsen Ltd., Salfords, Redhill United Kingdom at a crosshead speed of 2 mm/min. Five specimens were tested, per sample, at room temperature to obtain an average value of properties. Testing and data analysis were performed using Tinius Olsen Horizon software version 10.2.2.24. The flexural strength ($\sigma_{f}$) and flexural modulus ($E_{f}$) were calculated using the following standard Equations:

(4) $$\mathrm{Flexural}\;\mathrm{stress}:\;\sigma_{f}=\frac{3FL}{2bh^{2}}$$

where F is the maximum load, L is the support span, b is the width of the specimen, h is the thickness of the specimen. The average values of flexural strength and modulus for each sample were reported.

Following this, representative cross-sections of the manufactured laminate samples, with dimensions of 10 mm × 12 mm, were cut from each laminate type. The cross-sections were mounted, polished, and observed using an optical microscope (Olympus BX53M) supplied by EVIDENT Europe GmbH, United Kingdom. The microscope, equipped with a camera and the Olympus Stream imaging analysis software, was used to observe voids.

### 2.4. Thermo-Mechanical Analysis

The thermo-mechanical properties of the fabricated recycled composite material were evaluated utilising a Perkin Elmer DMA 8000 dynamic mechanical analyser, supplied by PerkinElmer, Buckinghamshire, United Kingdom. Specimens of rectangular geometry, measuring 17.5 mm × 10 mm × 3.7 mm, were subjected to flexural stress in a single cantilever configuration. The analysis was conducted over a temperature range of 25 °C to 250 °C, with a linear heating rate of 5 °C/min. Throughout the experiment, a constant frequency of 1 Hz was maintained, with an oscillation amplitude of 50 µm.

### 2.5. Fabrication and Characterisation of rCF-Based Capacitive Touch/Proximity Sensors

**Figure 2:**
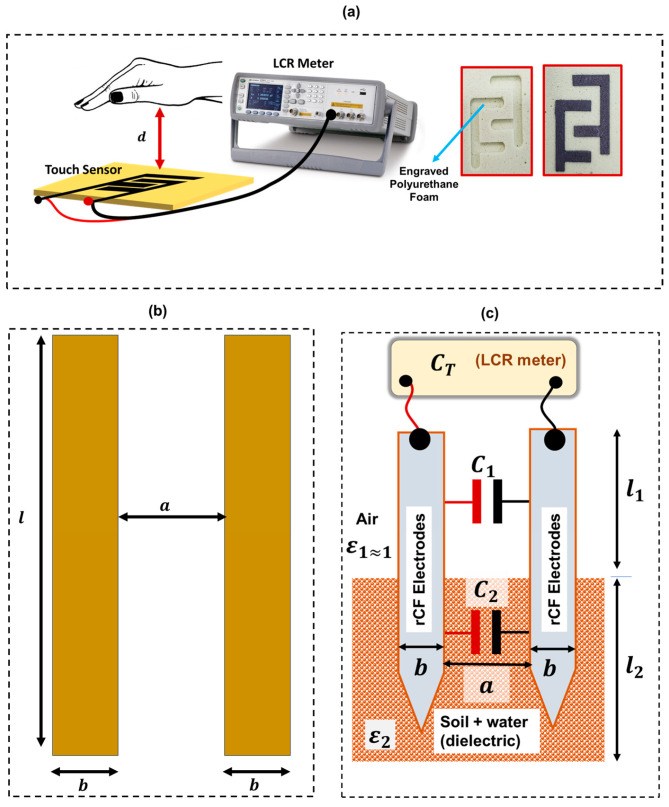
(**a**) Structure and working principle of the touch sensor (**b**) coplanar capacitor model (**c**) Structure and working principle of the soil moisture sensor.

After examining the structure of the rCF composites, described in Section 2.1, five similar touch sensors (TS-1, TS-2, TS-3, TS-4, and TS-5) were realised using sample 1 (Table 2). The sensors can measure both proximity and touch, with *d* representing the distance between the hand and the sensor. As *d* reduces, the capacitance of the sensor increases (Figure 2a).

To design the sensors, first, an interdigitated structure (width of electrode = 7 mm, spacing = 7 mm) was designed using a computer-aided design (CAD) application. The designed structure was engraved on a polyurethane foam substrate (Figure 2a) using a Computer Numerical Control (CNC) router machine. Following this, waterjet equipment was then used to cut out the electrodes from the fabricated rCF laminate. The rCF electrodes were then systematically fitted into the engravement, initially created on the polyurethane foam substrate, as shown in Figure 2a, to realise an interdigitated capacitive touch/proximity sensor.

Subsequently, an LCR meter was used to characterise the performance of the fabricated sensor by measuring its capacitance when approached by the hand or touched. For proximity sensing, measurements were carried out at three values of d (100 mm, 50 mm, and 20 mm) (Figure 2a). The response of the sensor was also randomly recorded for absolute touch (d = 0) to investigate their response to touch and the ability of the sensor’s output to return to its base value.

### 2.6. Fabrication and Characterisation of rCF-Based Soil Moisture Sensors

The fabricated rCF-based soil moisture sensor (SMS), realised in this work, uses the capacitive approach to measure the soil moisture content. Figure 2b,c shows the working principle and structure of the fabricated rCF-based SMS. Figure 2b shows two coplanar electrodes of length *l*. If the width of the electrode is *b*, and the spacing between the electrodes is *a*, then the capacitance, *C*, of the coplanar electrodes, with a dielectric material (dielectric constant = *ε*) between them, can be expressed, as shown in Equation (5) [40,41]. Equation (5) could also be used to estimate the capacitance of the SMS when it is fully inserted into the soil. However, if the soil moisture sensor is partially inserted, as shown in Figure 2c, then the capacitance, *C*_*T*_, of the SMS is given by Equation (6), which considers two different coplanar capacitors, *C*_1_ and *C*_2_, having a length of *l*_1_, and *l*_2_, respectively. In this case, air is the dielectric (dielectric constant = *ε*_1_) for *C*_1_, while soil, as well as its water content, is the dielectric (dielectric constant = *ε*_2_) for *C*_2_. Considering this, as the moisture content of the soil changes, *ε*_2_ also changes, causing a corresponding change in the capacitance *C*_*T*_.

In this work, three different capacitive soil moisture sensors (SMS-1, SMS-2 and SMS-3) were fabricated using recycled carbon fibre (rCF) of different fibre-to-epoxy ratios, as described in Section 2. For each sensor, two V-shaped electrodes (Figure 2c) were cut out of the fabricated rCF laminate. The tip of the sensor was made V-shaped to make it easy to penetrate the soil, while insulated crimp connectors were attached at the opposite ends to couple to wires. The connectors were reinforced using epoxy glue, and flexible wires were soldered onto the crimp connectors and then connected to an LCR to systematically read the output of the sensors.

(5) $$C_{T}=\frac{\varepsilon l}{\pi }ln\left(1+\frac{b}{a}\right)$$

(6) $$C_{T}=\frac{ln\left(1+\frac{b}{a}\right)(\varepsilon_{1}l_{1}+\varepsilon_{2}l_{2})}{\pi }$$

The characterisation of the sensors was carried out by inserting the sensors, in turn, into a dry soil sample, and the base capacitance measured using the connected LCR meter was noted. With the sensor still in the soil, different volumes of water (in steps of 2 mL) were carefully added to the soil sample using a graduated transfer pipette, and the output of the sensor was recorded in each case. Following this was a cyclic measurement, where the sensors were systematically dipped and removed from the soil to determine their stability.

## 3. Results and Discussion

### Characterisation of the rCF Sensing Electrode

**Figure 3:**
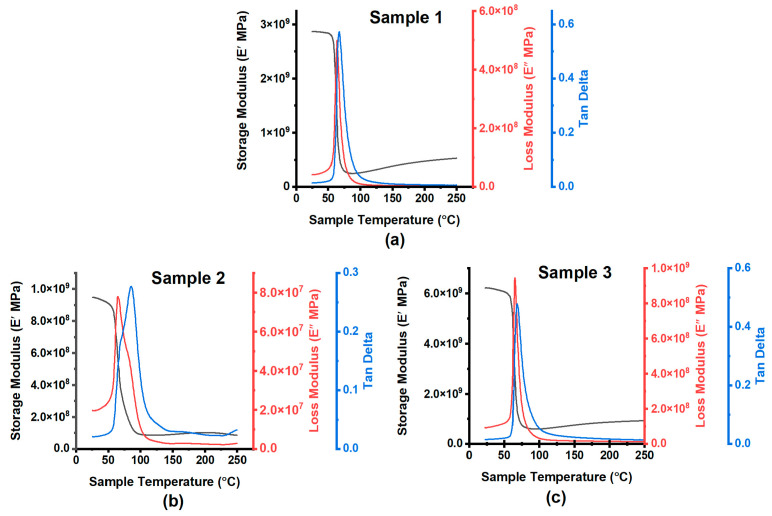
DMA plot for (**a**) Sample 1 (**b**) Sample 2 (**c**) Sample 3.

**Figure 4:**
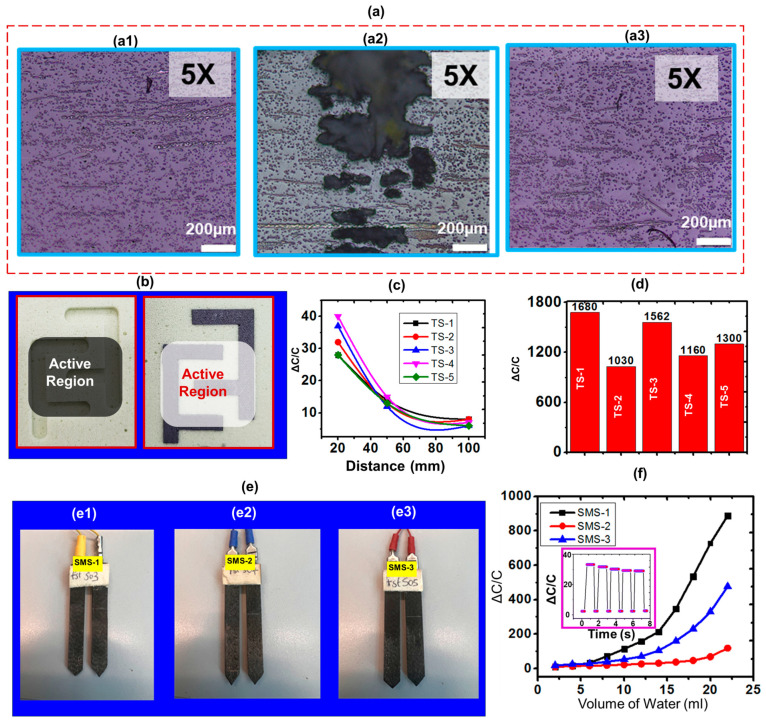
(**a**) Optical microscopic image of the fabricated rCF laminate: (**a1**) Sample 1, (**a2**) Sample 2, (**a3**) Sample 3, (**b**) fabricated touch sensor, (**c**) response of the five similar touch sensors to proximity, (**d**) response of the touch sensors to absolute touch, (**e**) image of the fabricated soil moisture sensors, (**f**) response of the three soil moisture sensors to soil of varying water.

The fibre volume fraction and void content were determined for the three manufactured composite samples, as summarised in Table 2. Sample 3 exhibited the highest fibre volume fraction at 17%, compared to 10% and 11% for Samples 1 and 2, respectively. The corresponding void contents were 2% for Samples 1 and 3 and 10% for Sample 2. Charts illustrating the relationships between the storage modulus, loss modulus, and tan delta for each of the tested specimens are shown in Figure 3. Further more, microstructural analysis via microscopy revealed the distribution of the randomised recycled carbon fibres within the epoxy matrix, as shown in Figure 4(a1)–(a3). Sample 3 showed a greater density of fibres compared to Samples 1 and 2, which correlates to the quantified fibre volume fractions. The higher void content of 10% for Sample 2 can be observed in Figure 4(a2) as increased porosity. With an increased fibre volume fraction of recycled carbon fibres, the randomised short carbon fibres begin to make intimate and continuous contact through the thickness of the composite. As a result, the composite becomes electrically conductive. Conversely, the presence and distribution of voids impede the continuity of fibre contacts, which reduces conductivity through the thickness of the composite. Electrically, the magnitude of fibre present in fibre-reinforced composites is directly proportional to the overall conductivity of the laminate [42].

The flexural properties of the rCF composites are presented in Table 3. The results showed that the flexural strength of the rCF samples varied over a range of values, from about 72 MPa to 231 MPa, while the average range for the flexural modulus was from about 6 GPa to about 10 GPa. These values are considered low for industrial/structural applications, as established by [43], but sufficient for the targeted sensing application.

The evaluated results from the thermomechanical analysis, performed on the rCF laminates, using DMA 8000, for sample 1, sample 2 and sample 3, are highlighted in Table 4. 

Figure 4a shows the optical image of the three different rCF composites (Sample 1, Sample 2 and Sample 3) fabricated with the compositions shown in Table 4. The results clearly show that sample 2 had more voids (Figure 4(a2)) compared to sample 1 (Figure 4(a1)) and sample 3 (Figure 4(a3)). Figure 4b shows the structure of the touch sensors and the active region. Figure 4c shows the result of the proximity sensing characterisation for the five similar touch sensors (TS-1, TS-2, TS-3, TS-4, and TS-5) fabricated. As expected, it clearly shows an increase in capacitance for a decrease in the distance of separation between the hand and the sensor, with a standard deviation of ~5.38 for 20 mm, 1.14 for 50 mm and 1 for 10 mm distance. For a distance of 20 mm, the average change in capacitance (ΔC/C) for all the sensors was ~34, and for 100 mm, it reduced to ~5. With a base capacitance of ~2.2 pF, the change in capacitance was observed to be relatively high, ~1.680 nF (ΔC/C~750), when the active regions of the touch sensors were touched with the fingers (Figure 4d). Figure 4e shows samples of the fabricated SMS, and Figure 4f shows the result of the three fabricated soil moisture sensors (SMS-1, SMS-2, and SMS-3). It shows an increase in capacitance, with an increase in the volume of water added, and with a maximum sensitivity of ~116 pF/mL of water recorded for SMS-1 in the linear region (Figure 4f). 

The inset in Figure 4f shows the cyclic response of the sensor, which shows that the sensor is stable. For all measurements, the sensor was able to return to its base capacitance of ~2.2 pF. The electrodes of the sensor were also immersed in water and left for 10 days, and no degradation was observed. Only the base capacitance was seen to slightly increase from ~2.2 pF to ~2.3 pF. From Figure 4f, SMS-1 was seen to have a better performance and SMS-2 the least. This could be attributed to their composition (Table 1) and structure (Figure 4a). SMS-1 contains more fibre and less epoxy compared to SMS-2 and SMS-3 and also has more evenly distributed fibre content within the matrix. On the other hand, SMS-3 has a number of voids in its structure, as can be seen in Figure 4(a2).

## 4. Discussion

This study analysed three rCF composite samples with varying fibre volume percentages and void contents. Sample 1 contained 10% fibre volume with 2% void content, Sample 2 had 11% fibre volume with 10% void content, and Sample 3 included 17% fibre volume with 2% void content. These variations in composite content directly influenced the mechanical and sensing properties of the samples. The optical microscopic images in Figure 4a further illustrate the structural integrity and homogeneity of the rCF laminates. Sample 1 and Sample 3, with lower void contents, appeared more uniform compared to Sample 2, as provided in a qualitative assessment of the rCF composite laminates’ microstructure. These images offer insights into the fibre distribution, alignment, and potential defects within the composite samples.

The flexural properties measured for these samples displayed significant differences. Sample 1 exhibited a flexural strength of 231.42 MPa and a flexural modulus of 997 GPa, indicating robust mechanical performance. Sample 3, with a higher fibre volume percentage, also showed high flexural strength at 219.35 MPa, although its flexural modulus was lower at 9.86 GPa compared to Sample 1. Sample 2, with the highest void content, demonstrated the lowest flexural strength (71.69 MPa) and modulus (6.08 GPa), underscoring the detrimental impact of high void content on the mechanical integrity of the composites. These results align with existing literature, which suggests that increased fibre volume typically enhances mechanical properties, while higher void content adversely affects strength and stiffness [44].

The results of the dynamic mechanical analysis (DMA), presented in Figure 3a–c, provide insights into the viscoelastic behaviour of the rCF composites under dynamic loading conditions. As expected, the glass transition temperatures (Tg) of the three samples fell within a relatively narrow range (66.5 °C to 67.5 °C), which is characteristic of standard epoxy resin. This slight variation in Tg could be attributed to the influence of void. Further analysis substantiated these findings, revealing that the mechanical behaviour of the rCF composites is significantly influenced by their internal structure and composition. Sample 3, with the highest fibre volume percentage, exhibits the greatest stiffness. In contrast, Sample 2, which has the highest void content, shows the lowest storage modulus, indicating reduced stiffness. Sample 1 presents moderate stiffness, balancing flexibility and robustness. These variations suggest differences in the microstructural adhesion of the composites, potentially due to factors such as fibre volume fraction or the quality of fibre-matrix interfacial bonding [45].

The results of the loss modulus (E) show that Sample 2, characterised by a broader curve, has the highest internal friction. This is likely due to its higher void content, leading to greater energy dissipation. Sample 3, despite its high fibre content, does not significantly increase energy dissipation, maintaining a moderate loss modulus. Sample 1, again, shows balanced properties with lower internal friction. The results of the damping factor (tan δ) suggest that Sample 1, with the highest tan δ, offers better damping capabilities, making it suitable for environments with significant mechanical vibrations. Sample 3, with a lower tan δ but higher stiffness, provides a good balance between damping and rigidity, while Sample 2 maintains moderate damping properties. These thermo-mechanical properties could directly impact the performance and reliability of the capacitive sensors.

The results of the touch/proximity sensing show an average change in capacitance (ΔC/C~34) for 20 mm and (ΔC/C~5) for 100 mm distance of a hand from the active sensing region. This sensitivity is significant, indicating that rCF composites can effectively function as touch and proximity sensors. The proximity sensing results are comparable to those found in studies on carbon-based capacitive sensors, which often highlight the high sensitivity and rapid response times of these materials [46].

The soil moisture sensors demonstrated stable and repeatable responses, with a high sensitivity of approximately 116 pF/mL of water in the linear region. This performance suggests their applicability in smart agricultural systems, where accurate soil moisture monitoring is critical. The results are in line with existing research on capacitive soil moisture sensors, which emphasises the importance of high sensitivity and repeatability for effective agricultural monitoring [47].

The study’s findings underscore the potential of rCF composites in developing sustainable and efficient sensor technologies. The use of recycled materials aligns with current trends in environmental sustainability and circular economy, providing a dual benefit of reducing waste and creating high-performance sensor systems. This approach is supported by literature advocating for the integration of recycled materials in advanced applications to promote sustainability without compromising functionality [48,49].

Moreover, the mechanical and electrical properties of rCF composites can be tailored by adjusting the fibre volume and void content, offering flexibility in designing sensors for specific applications. Unlike some conventional sensors [50], where the simultaneous tuning of mechanical and electrical properties is limited or impossible, our rCF-based sensors have the potential to be used in applications (e.g., automobile, human–machine interfaces, etc.) where varying stiffness of materials are used to develop different parts of the system, depending on their functionality. This means that it creates the opportunity for the integration of intrinsic sensing into passive structures having varying stiffness. The high flexural strength and modulus of certain samples indicate that these materials can withstand significant mechanical stress, making them suitable for applications requiring durability. This is advantageous when compared to reported sensors [51] realised using recyclable materials, such as paper and flexible polymers, which are not durable [17,23]. Such disposable sensors are mainly beneficial for applications requiring single use. The sensitivity of the touch and proximity sensors highlights their potential in human-machine interfaces, where quick and accurate touch detection is essential [52].

In summary, the experimental results present rCF composites as promising materials for capacitive sensing applications. Despite rCF having some limitations for sensing applications, such as manufacturing challenges and integration with electronic circuits, their mechanical properties, touch/proximity sensing capabilities, and soil moisture monitoring performances are competitive with existing sensor technologies. By leveraging recycled materials, these sensors not only contribute to sustainability but also offer high performance, aligning with the growing demand for eco-friendly and efficient sensor solutions.

## 5. Conclusions

In this work, capacitive sensors have been realised using recycled carbon fibre (rCF) composites. In particular, the fabricated composite was used to realise capacitive touch sensors and soil moisture sensors, demonstrating the use of the rCF for the fabrication of sensors for such applications. Using the rCF as an electrode for the soil moisture sensor, in particular, has a sustainability advantage as it eliminates the issue of electrode corrosion present in conventional soil moisture sensors, which are made of metal electrodes. The performance of both categories of capacitive sensors (touch sensor and soil moisture sensors) shows a high change in capacitance, and this potentially opens more research opportunities for touch sensing in automobiles and the development of sustainable sensors for smart agriculture. The experimental results from the study on rCF-based capacitive sensors demonstrate their high sensitivity, stability, and suitability for touch/proximity sensing and soil moisture detection. These findings are consistent with existing literature on capacitive sensors, confirming the potential of recycled carbon fibre composites for the development of efficient and sustainable sensor technologies. Future research should focus on optimising the manufacturing processes to further reduce void content and enhance the performance of these sensors, as well as exploring their applications in other fields, such as wearable electronics and environmental monitoring. The integration of rCF composites in sensor technology not only provides a viable solution for electronic sensing applications but also contributes to environmental sustainability by promoting the recycling and reuse of carbon fibre materials. Future work could also explore the realisation of self-powered sensors using rCF and the combination of other materials with rCF composites for multifunctional sensing.

## Figures and Tables

**Table 1:** Comparison of existing soil moisture sensors.

Type of Electrode	Sensing Mechanism	Reference
Copper	Capacitive	[29]
Gold	Resistive	[31]
Gold	Resistive	[32]
Copper	Capacitive	[33]
Graphene Oxide	Capacitive	[34]
Stainless Steel	Resistive	[30]

**Table 2:** rCF composite content analysis.

Sample No.	Fibre Volume Percent (%)	Void Content (%)
Sample 1	10.00	2.00
Sample 2	11.00	10.00
Sample 3	17.00	2.00

**Table 3:** Flexural properties of rCF composite laminate.

Property	Specimen ID		
	Sample 1	Sample 2	Sample 3
Flexural Strength (MPa)	231.42	71.69	219.35
Flexural Modulus (GPa)	9.97	6.08	9.86

**Table 4:** Dynamic Mechanical Analysis of rCF Composite.

Sample No.	Storage Modulus (MPa) @ 25 °C	Loss Modulus (Peak) (MPa)	Tan Delta (Peak)	Glass Transition Temperature, T_g_ (°C)
Sample 1	$2.9\times 10^{9}$	$4.9\times 10^{8}$	0.57	66.5
Sample 2	$9.5\times 10^{8}$	$7.7\times 10^{7}$	0.27	62.4
Sample 3	$6.2\times 10^{9}$	$9.4\times 10^{9}$	0.48	67.5

## Data Availability

Data are contained within the article.
